# Operation for Appendicitis

**Published:** 1896-01

**Authors:** W. O. Roberts

**Affiliations:** Professor of Surgery and Clinical Surgery in the University of Louisville, Louisville, Ky.


### OPERATION FOR APPENDICITIS.

REPORTED TO THE LOUISVILLE SURGICAL SOCIETY.

BY W. O. ROBERTS, M. D.,
Professor of Surgery and Clinical Surgery in the University of Louisville, Louisville, Ky.

The specimen which I show you is from a young man about twenty years of age, that I saw for the first time yesterday afternoon with Dr. Cottell. The history was that the young man was seized with sudden, severe pain in the abdomen three days ago, and was seen by a physician, who administered an opiate for the relief of pain. When Dr. Cottell saw him, pain was still severe; there was no elevation of temperature, but a very frequent pulse; excessive vomiting, no action of the bowels; no marked tympanites.

When I was called in consultation, yesterday afternoon, there was no elevation of temperature, patient still vomiting, no passage of gas or fæcal matter from the bowels, pulse exceedingly feeble and 138 to the minute. We had him removed to the Norton Infirmary, and upon his arrival there, his pulse had not materially changed, very little distension of the abdomen, but intense pain, chiefly referred to the umbilical region. No tumor could be detected. He had urinated, so he said, early in the morning, but could not do so that afternoon. I had him catheterized and drew off two ounces of urine, which contained some albumen. His tongue was very dry. He had been given a dose of morphine hypodermatically at ten o'clock in the morning, since which no medicine of any kind had been administered. We decided to do a laparotomy, and upon opening the abdomen at the usual point for appendicitis, a quantity of pus poured out. It was found there was pus throughout the peritoneal cavity; the appendix was ruptured at its base; it was removed, the cavity thoroughly cleansed, and a drainage tube inserted. His pulse improved after the operation, and was 128 to the minute this morning. He has not vomited since the operation, but the secretion of urine has been very scanty. Temperature, this morning, $97\frac{4}{5}$ F.; this afternoon, temperature 100, pulse 128.

The interesting point in this case, to me, is that the man has not had a febrile temperature during the trouble, and there has been no marked distension of the abdomen.

REMARKS.

DR. A. M. CARTLEDGE: It is very likely that the patient had a febrile temperature before a physician was called. It is probable that an appendiceal abscess ruptured without adhesions, and we know, coincident with such rupture, the temperature often falls. The same thing occurs when we have formation of an abscess which ruptures within the peritoneum. After absorption of ptomaines in septic peritonitis, for instance, the temperature often falls to subnormal, which makes these cases very deceptive; they seem much better, after rupture occurs, for a short time. A unique feature in the case reported is that the patient had no tympanites. Another interesting point is the prognosis. I think we can make a reasonable prognosis, if we can judge from the history of the case when rupture of the appendix occurred. If over twelve hours elapsed between rupture of appendix with escape of pus into the peritoneal cavity, and the operation, the patient will probably die. It depends upon the amount of absorption which has taken place. If operation is performed within twelve hours after rupture, and the cavity is thoroughly cleansed, our patient stands a good chance of getting well. I believe this is the dividing line in making a prognosis.

---

BLOODLESS OPERATION FOR HÆMORRHOIDS.—Manley (*Boston Medical and Surgical Journal*) describes his bloodless method of treating hæmorrhoids. Before operating, two to four ounces of whisky are administered, and effective cocainization applied hyperdermically. Anal dilatation, gradual and steady, without rupture of the muscle, is done, and after drying and mopping with cocaine solution, each hæmorrhoid is separately seized close to its base firmly between the tip of the thumb, index and middle fingers. It is put on full stretch, then twisted, and finally, so completely crushed that it is reduced to a pulp, and none of the investing tunics remain except the mucous membrane and its under stratum of fibrous tissue. The mass is then returned and an opium suppository introduced. He has treated thirty-five cases in this way with perfectly satisfactory results.—*St. Louis Medical and Surgical Journal.*

